# A Case Report of Aerococcus urinae Urinary Tract Infection in an Elderly Male with Multimorbidity

**DOI:** 10.7759/cureus.26379

**Published:** 2022-06-27

**Authors:** Bailey Balouch, Salina Munankami, Ayushma Acharya, Manish Shrestha, Swarup Sharma Rijal

**Affiliations:** 1 College of Medicine, Drexel University College of Medicine, Philadelphia, USA; 2 General Medicine, Kathmandu Medical College, Kathmandu, NPL; 3 General Medicine, Helping Hands Community Hospital, Kathmandu, NPL; 4 Internal Medicine, Reading Hospital, Reading, USA; 5 Internal Medicine, Reading Hospital - Tower Health, Reading, USA; 6 Internal Medicine, Tower Health Medical Group, Wyomissing, USA

**Keywords:** symptomatic uti, urinary tract infection (uti), prostate cancer, antibiotics therapy, aerococcus urinae

## Abstract

*Aerococcus urinae* is a rare cause of urinary tract infection (UTI) seen in elderly males with multimorbidity. Incidence is estimated between 0.15 and 0.8%. This organism is frequently misidentified for other gram-positive species. Missed or delayed diagnosis of *A. urinae* UTI can lead to systemic infection with high morbidity and potential mortality. We present a classic case of *A. urinae* UTI in a 91-year-old male with multiple comorbidities, including heart failure, diabetes mellitus, and metastatic prostate carcinoma. Empiric therapy with nitrofurantoin was unsuccessful, but intravenous ceftriaxone and bladder catheterization resulted in rapid symptomatic improvement. Variable antimicrobial sensitivities and resistance have been reported for *A. urinae*. Therefore, antimicrobial resistance testing should be performed for all patients with *A. urinae* infections.

## Introduction

*Aerococcus urinae* is an uncommon organism that has, in rare cases, been linked to urinary tract infections (UTI), particularly in elderly male patients with anatomic urinary tract abnormalities such as those seen with urologic cancer [1,2]. The gram-positive, coccoid, *A. urinae* species was not defined until 1992, and since then, the incidence of *A. urinae* UTI has been estimated at 0.15-0.8% [3,4]. The true incidence of *A. urinae* UTI is likely severely underestimated as it can frequently be misidentified as Staphylococcus, Streptococcus, or Enterococcus species [1,2,4]. Patients who delay treatment or have a strain of *A. urinae* resistant to the antibiotic they are receiving are at increased risk of progressing to systemic invasion. Several reported cases have been notified of *A. urinae* bacteremia, endocarditis, and spondylodiscitis [5-10]. Early recognition of this rare fastidious organism is critical to preventing severe illness and death. We here describe a classic *A. urinae* UTI in an elderly male patient with metastatic prostate carcinoma.

## Case presentation

**Figure 1:**
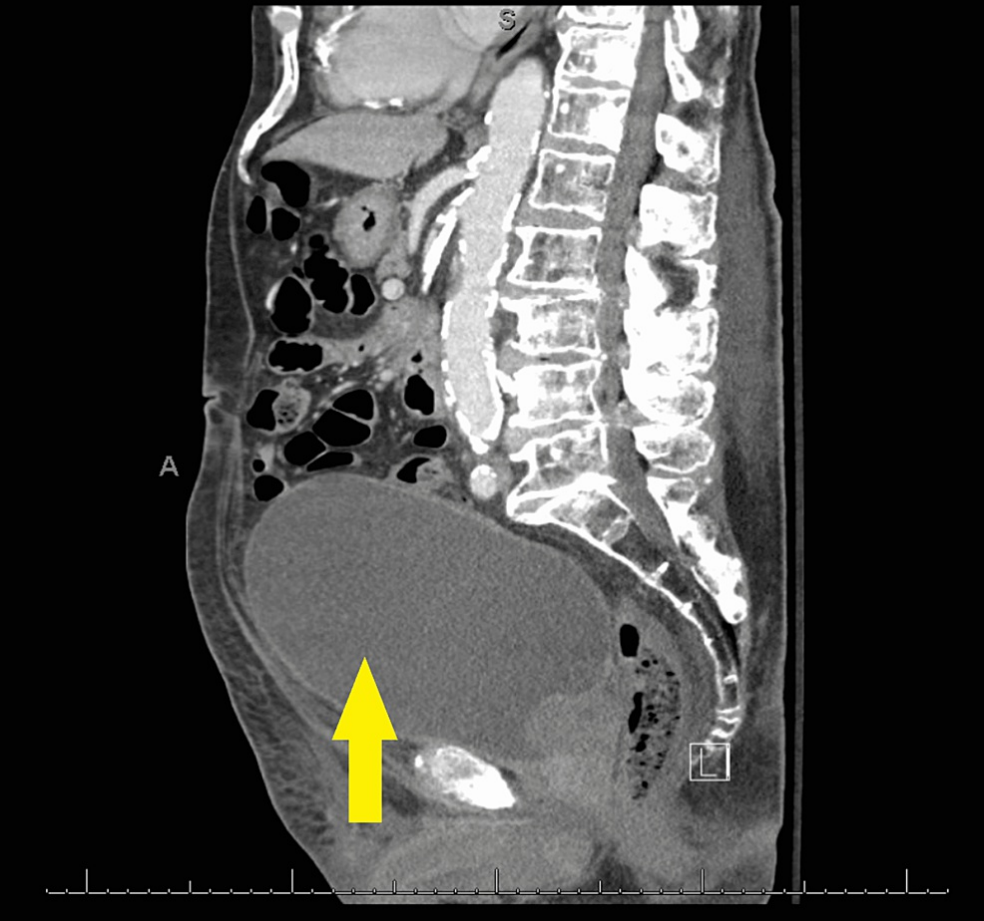
Markedly distended urinary bladder (Yellow arrow).

**Figure 2:**
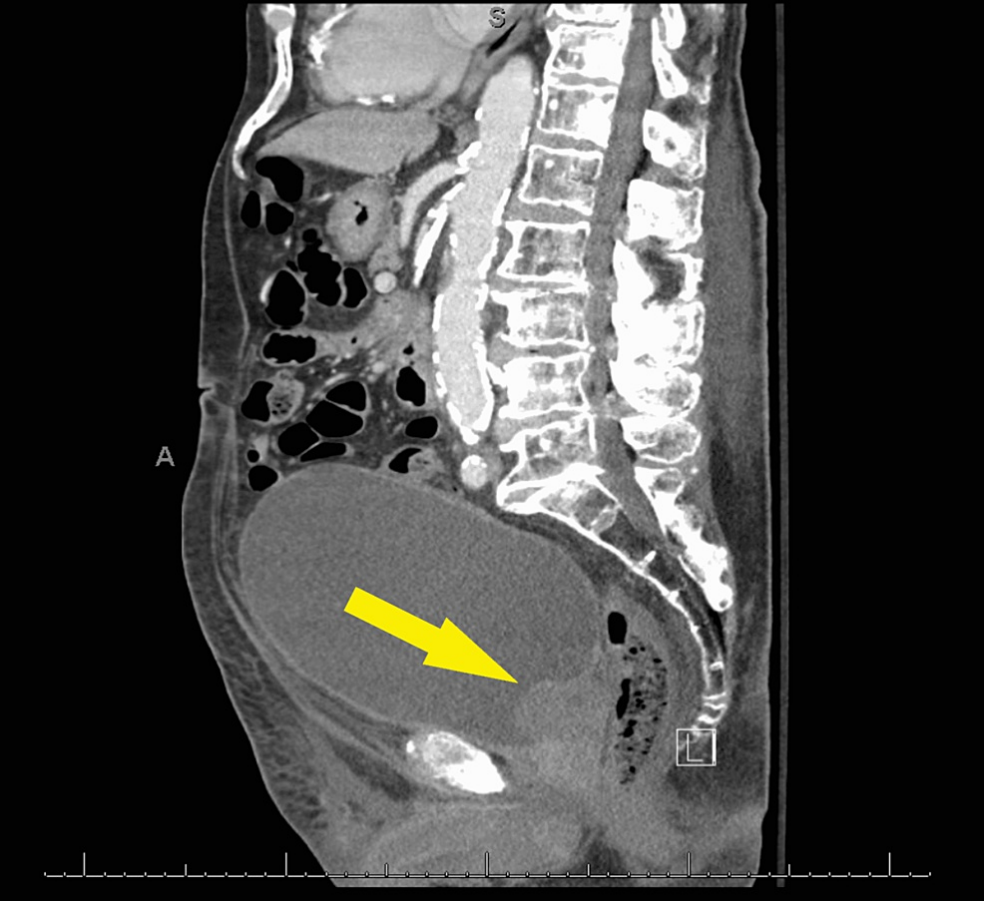
Enlarged prostate gland (Yellow arrow).

**Figure 3:**
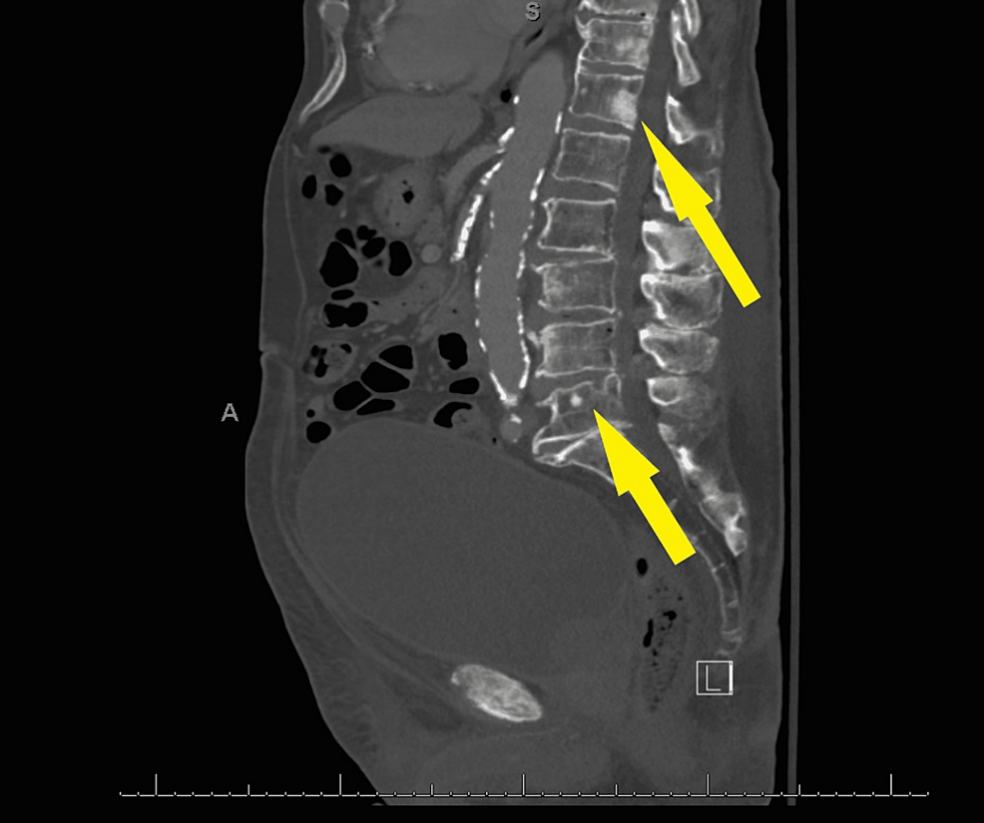
Sclerotic bony densities in the spine consistent with metastatic prostate carcinoma (Yellow arrows).

A 91-year-old male presented to the urgent care clinic with a complaint of dysuria for three days associated with urinary frequency and urgency, but without fever, chills, flank pain, or hematuria. He had a medical history significant for benign prostatic hyperplasia, spinal stenosis, heart failure with improved ejection fraction, type 2 diabetes mellitus, and prior hospitalization for Fournier’s gangrene eight years back. Point of care urinalysis at presentation demonstrated cloudy yellow urine with small leukocyte esterase but no nitrite, no blood, 30 mg/dL protein, pH of 6.5, and specific gravity of 1.030. Urine was sent for culture, and the patient was started empirically on oral nitrofurantoin. Symptoms had not improved by day two of antibiotic therapy, and cultures returned with >100,000 CFU/mL *A. urinae* in addition to mixed gram-positive organisms in lesser quantity. *A. urinae* was identified from urine cultures biochemically via a phenotypic approach utilizing a commercially available reagent card system. Although newer methods are available for the detection of *A. urinae*, they are not used routinely in our institution. Unfortunately, antimicrobial sensitivity testing was not performed. The patient was contacted and advised to stop the nitrofurantoin and start on a 7-day course of oral cephalexin. Before the patient could switch antibiotics, he presented to the emergency department with a chief complaint of low back pain and difficulty with ambulation. Urine culture was not repeated in this presentation. The back pain was similar to that he had experienced in the past due to his spinal stenosis but had worsened in severity over several months. He also endorsed urinary retention and progressively worsening large-volume urinary incontinence. The patient was afebrile and hemodynamically stable. Straight catheterization produced 800 cc of urine. Repeat urinalysis revealed cloudy dark yellow urine with large blood, trace ketones, 30 mg/dL protein, pH of 6.0, and specific gravity of 1.016. Urinalysis was negative for leukocyte esterase and nitrite. There were 154 RBCs per high-power field (HPF), zero epithelial cells per HPF, three WBCs per HPF, and many bacteria. Computed tomography imaging of the abdomen and pelvis with contrast demonstrated a markedly distended urinary bladder (Figure 1), moderate bilateral hydroureteronephrosis, enlarged prostate gland (Figure 2), and sclerotic bony densities in the spine consistent with metastatic prostate carcinoma (Figure 3). The patient was started on intravenous ceftriaxone to manage the *A. urinae* UTI. Several attempts were made to place a Foley catheter but were unsuccessful. A Foley catheter was placed the following day successfully, and >1100 cc of urine was produced. His urinary symptoms improved rapidly.

## Discussion

We here describe a classic *A. urinae* UTI in an elderly male patient with metastatic prostate carcinoma. Advanced age and multimorbidity are well-described predisposing factors for *A. urinae* infection. Urinary retention, other urologic diseases including benign prostatic hyperplasia, prostate, bladder, or colon malignancy, long-term indwelling catheter placement, heart disease, diabetes, and chronic kidney disease have all been cited as comorbid conditions that increase susceptibility [4,10,11]. Healthcare exposures should also be considered a risk factor [12]. *A. urinae* may be more common in males, but some authors suggest that both sexes are affected equally [1,4,10]. In one study of 16 patients with *A. urinae* UTI, 93.75% were males over 70 [10]. Males appear at greater risk for bacteremia and severe complications from underlying *A. urinae* UTIs [2]. Patients with *A. urinae* UTI present initially with mild localized symptoms such as urinary frequency, urgency, dysuria, nocturia, and difficulty voiding with or without hematuria [1,4,13]. In this case report, the patient presented with urinalysis positive for blood, protein, and esterase but negative for nitrites, as has been previously reported.

*A. urinae* are gram-positive cocci commonly found in clusters, pairs, or tetrads. They are microaerophilic, grow on 6.5% NaCl agar, and produce alpha-hemolysis on 5% horse blood agar [1,3]. Diagnosis of *A. urinae* infection may require genome sequencing, as this organism is frequently misidentified as other gram-positive species such as Streptococci, Staphylococci, or Enterococci [1,2,4,12].

Colony morphology has been noted to be similar to that of viridans streptococci [14]. Therefore, proper identification and determination of antibiotic susceptibilities are critical to preventing sequelae of *A. urinae* UTIs [1].

*A. urinae* has shown increasing resistance to vancomycin and penicillin, in particular [14]. However, some authors have noted that strains isolated from their patients were resistant to sulfonamides, fluoroquinolones, macrolides, and clindamycin [2,4,15]. In some cases, *A. urinae* has responded well to fluoroquinolones, amoxicillin, piperacillin/tazobactam, and ampicillin/gentamycin [1,6,16]. In vitro, *A. urinae* was susceptible to amoxicillin, cefotaxime, ceftriaxone, doxycycline, linezolid, meropenem, penicillin, rifampin, trimethoprim-sulfamethoxazole, and vancomycin in one study [15]. In our patient, the laboratory recommended antimicrobial therapy with penicillin, tetracycline, cefazolin, or clindamycin. We chose to use cephalosporin and treated the patient successfully with ceftriaxone. Initially, we started empirical nitrofurantoin with poor symptomatic response to treatment. Antibiotic sensitivity is not consistent between strains of *A. urinae*, so antimicrobial sensitivity testing should be performed for all patients with *A. urinae* UTI.​​​​​​ Clinical characteristics of patients with *A. urinae* and antibiotics susceptibilities from different studies are listed in Table 1.

**Table 1:** Clinical characteristics of patients with Aerococcus urinae and antibiotics susceptibilities [4].

Author, year	Number of cases	Comorbidities	Treatment/Susceptibilities
Zhang et al., 2000 [1]	2	Coronary artery disease, cerebrovascular accident, dementia, hypothyroidism	Ciprofloxacin, tetracycline
Sierra-Hoffman et al., 2005 [17]	32	Diabetes, renal disease, heart disease, institutionalization, urologic disease, urinary catheter	Ceftriaxone, levofloxacin, penicillin, tetracycline, ad vancomycin
Senneby et al., 2012 [10]	16	Cerebrovascular accident, chronic lymphocytic leukemia, chronic obstructive pulmonary disease, colon cancer, dementia, ischemic heart disease, myelodysplastic syndrome	Amoxicillin, cefadroxil, cefuroxime, cefotaxime, ciprofloxacin, clindamycin, erythromycin, gentamicin, imipenem, levofloxacin, Meropenem, penicillin, piperacillin-tazobactam
Rasmussen, 2013 [2]	24	Alcoholism, aortic stenosis, atrial fibrillation, atrial septal defect, chronic obstructive pulmonary disease, colon cancer, congestive heart failure, dementia, diabetes mellitus, Down’s syndrome, ischemic heart disease, mitral regurgitation	Amoxicillin, ceftriaxone, cefotaxime, clindamycin, fosfomycin, penicillin, piperacillin, vancomycin
Senneby et al., 2014 [18]	64	N/A—epidemiology study	Ampicillin, cefalotin, ciprofloxacin, mecillinam

*A. urinae* remains an abstruse organism with poor awareness among healthcare professionals despite the great morbidity and mortality associated with complicated UTIs from this organism, particularly when a systemic invasion occurs. Although several cases have reported severe manifestations of complicated UTI, few have focused on the early diagnosis prior to systemic infection. Our case highlights the risk factors associated with *A. urinae* UTI and serves to remind physicians that *A. urinae* initially presents as a seemingly uncomplicated UTI and reinforces our understanding of the importance of timely diagnosis and treatment of this organism.

## Conclusions

*A. urinae* is a rare cause of UTI usually seen in elderly patients with multimorbidity. It is essential for clinicians to be aware of *A. urinae *and to keep this pathogen on their differential, particularly for patients who may be at greater risk for *A. urinae* UTI and systemic infection. Differential antimicrobial resistance between strains warrants antimicrobial sensitivity testing for all patients with positive *A. urinae* cultures.
